# Large animal models for the assessment of snakebite envenoming therapies

**DOI:** 10.1038/s44386-026-00043-8

**Published:** 2026-04-01

**Authors:** Melisa Benard-Valle, Shirin Ahmadi, Cassandra M. Modahl, Edgar Neri-Castro, Alejandro Alagón, Leslie Boyer, Anne Ljungars, Andreas H. Laustsen

**Affiliations:** 1 Department of Biotechnology and Biomedicine, Technical University of Denmark, Kongens, Lyngby, Denmark; 2 Centre for Snakebite Research and Interventions, Liverpool School of Tropical Medicine, Liverpool, UK; 3 Departamento de Medicina Molecular y Bioprocesos, Instituto de Biotecnología, Universidad Nacional Autónoma de México, Morelos, México; 4 Ophirex, Inc., Corte Madera, California, CA USA; 5 University of Arizona, Tucson, AZ USA

**Keywords:** Biological techniques, Biotechnology, Drug discovery, Medical research

## Abstract

Snakebite envenoming causes over 100,000 deaths annually, creating a need for more effective therapies. Traditionally, most preclinical testing relies on murine models with limited translational value. This review highlights the value of large animal models, particularly sheep and pigs, for studying venom toxicokinetics and antibody and small-molecule pharmacokinetics. Implementing clear guidelines and standardized endpoints in large-animal studies could help advance the clinical translation of new snakebite treatments.

## The need for translational animal models in the development of envenoming therapies

Snakebite envenoming continues to claim hundreds of thousands of lives and limbs annually, predominantly in low- and middle-income countries in tropical regions of the world^1^. Currently, the only available specific therapeutic option against envenoming is plasma-derived antivenoms, consisting of polyclonal antibodies sourced from immunized animals^2^. While these medicines are essential for snakebite envenoming treatment and have saved countless lives, they suffer from several drawbacks due to their manufacturing process. These shortcomings include a low proportion of therapeutically relevant antibodies, limited neutralization across species, a propensity to elicit immunological adverse reactions, the necessity for parenteral administration by skilled healthcare personnel, and a relatively high cost of manufacture^3^. With the advances made in modern biotechnology and medicinal chemistry, there is now an opportunity to develop new types of envenoming therapies that potentially overcome the disadvantages of existing antivenoms^4–8^.

Historically, plasma-derived antivenoms have relied heavily on murine models for their assessment and quality control. These models have been invaluable in early-stage development, as snake venoms exert similar enzymatic and tissue-specific effects across mammals, allowing mice to provide important insights into the general efficacy of candidate treatments. However, the small size of mice imposes significant limitations. Individual animals cannot provide serial blood samples necessary for the efficient translation of pharmacokinetic and metabolic or hematologic endpoints to human patients. In addition, differences in absorption, distribution, metabolism, and excretion (ADME) between mice and humans further complicate the modeling of therapeutic dosing and duration. Consequently, while murine models remain important for initial pre-clinical testing, large animal models are essential to capture clinically relevant pharmacology and to bridge the gap toward human application^9,10^.

In this review, we use the term “large animal model” to denote non-rodent mammalian models whose body weight, plasma volume, and cardiovascular physiology are close enough to humans to generate translationally relevant data for the development of novel therapeutics, as compared to smaller animal models, such as mice, rabbits, or guinea pigs. Large animal models also allow clinically relevant procedures that cannot be performed in small animals, such as serial sampling, surgical interventions, and physiological instrumentation studies. Although this category technically includes species, such as goats and non-human primates, we focus on the two models most widely used in the field: ovine (sheep; *Ovis aries*) and porcine (pigs; *Sus domesticus*). Sheep are particularly valuable for studying venom toxicokinetics and antivenom pharmacokinetics, including lymphatic absorption^11–13^, whereas porcine models more closely recapitulate human skin, musculoskeletal anatomy, and local tissue pathology, enabling assessment of dermonecrosis, compartment syndrome, and clinically relevant treatment strategies^11,14^. The adequate use of these animal models is especially important for the development of new types of snakebite envenoming therapies, such as recombinant antivenoms and small molecule inhibitors, for which the traditional reliance on murine models poses a significant challenge. While both types of therapeutics have established utility in other areas of medicine, they lack the long clinical history of plasma-derived antivenoms in snakebite treatment, making it difficult or even impossible to predict their pharmacology for this indication^15,16^.

Even though the pharmacology of snakebite indications is not yet fully understood, ongoing preclinical progress suggests that new types of snakebite envenoming therapies are slowly getting closer to clinical implementation^17^. In particular, significant progress has been made in the development of broadly-neutralizing monoclonal and oligoclonal antibodies^4,7,18–21^, single-domain antibodies from camelids (i.e., nanobodies or V_H_Hs)^8,22–24^, and computationally designed minibinders^5^, as well as in the repurposing of small-molecule inhibitors with existing data from clinical trials^25–29^. It is hypothesized that these new treatment modalities offer potential advantages over plasma-derived antivenoms, and target product profiles have recently been proposed to facilitate their development into actual antivenom products^30^. In particular, recombinant approaches allow for the careful design of oligoclonal mixtures of broadly-neutralizing antibodies and V_H_Hs, which could be developed to have increased neutralization capacity and broader species coverage^24,31^. In parallel, orally bioavailable small molecule therapies, if sufficiently safe, may enable treatment to be initiated hours before parenteral antivenom administration becomes possible, *i.e*., treatment can be initiated while the patient might be enroute to a healthcare facility. Recombinant approaches allow for optimization of the pharmacokinetic and distribution properties of antivenoms, including half-life extension or enhanced deep tissue penetration^3^. The latter might enable faster toxin neutralization and thereby improved treatment of local tissue damage, a clinical outcome poorly addressed by existing antivenoms^1,8^.

While some of the abovementioned benefits of recombinant antivenoms and repurposed small molecule inhibitors have been demonstrated in murine models, their translational value remains largely unknown. The translation from murine models to larger animal models, let alone clinical application, is yet to occur for recombinant antivenoms, whereas some small molecule inhibitors, like varespladib, have been tested in larger animal models and in human snakebite victims^32,33^. Furthermore, one of the drawbacks of existing murine models is that neutralization of venom-induced lethality is evaluated as the main or only experimental endpoint. Other key clinical parameters, such as the prevention of local tissue damage, correction of coagulopathy, reversal of neurotoxicity, and support for tissue regeneration, are not assessed^34^. In addition, pharmacokinetics, toxicokinetics, and dosing are challenging to address in murine models and have limited translational applicability to clinical envenoming. In contrast, large animal models, *i.e*., animals with a body weight close to that of a human (such as sheep or pigs), may better mimic the pharmacokinetics and clinical outcomes of snakebite envenoming therapies in humans. Although methods for pharmacokinetic modeling exist for other pharmaceuticals, these have rarely been applied in the study of envenoming^13,35,36^. The limited use of large animal models highlights an ongoing challenge in bridging the gap between preclinical evaluation and clinical relevance for snakebite envenoming therapies.

This review aims to summarize the current knowledge derived from large animal models in assessing both plasma-derived and recombinant snakebite antivenoms, as well as snakebite therapeutics based on small-molecule inhibitors. By identifying knowledge gaps and highlighting areas for methodological refinement, we aim to facilitate the translation of novel therapeutics into clinical practice and improve outcomes for snakebite envenoming patients worldwide.

## Toxicokinetics of snake venom

Snake venoms are complex mixtures of toxins that contain a wide diversity of protein-based molecules, which vary significantly in molecular weight and biological function^37–39^. Following a snakebite, the injected venom may be delivered subcutaneously, as is common with small elapids, such as coral snakes, or intramuscularly, as sometimes observed with larger snakes, including cobras, mambas, or vipers. Once deposited, the venom is dispersed into the interstitial space, where small toxin molecules (under 16 kDa) are predominantly absorbed into circulation via the capillary system, while larger toxin molecules (above 100 kDa) are predominantly absorbed through the lymphatic system^36,40^. Lymph-borne toxins eventually enter systemic circulation through the thoracic duct, which allows the toxins to reach specific target sites^36,40–42^.

Sheep are particularly useful for studying the absorption of macromolecules and the role of the lymphatic system, as they allow lymphatic cannulation and continuous lymph collection, enabling direct quantification of the fraction of the drug absorbed via the lymphatic route. Such experiments are not feasible in small animal models, such as mice or rats, because sustained lymphatic cannulation is technically impossible, and the lymph volumes are too small for adequate sampling and analysis. In addition, the sheep lymphatic system exhibits anatomical and physiological characteristics that closely resemble those of humans, particularly in terms of lymph flow, vessel size, and macromolecule transport capacity, making it a relevant model for translational studies on lymphatic absorption^13,36,40,43,44^.

As an example of the distribution of small toxins, such as phospholipases A_2_ (PLA_2_s) and three-finger toxins (3FTxs), Paniagua and collaborators demonstrated that approximately 25% of the absorption of the venom of *Micrurus fulvius* occurs via the lymphatic route when administered subcutaneously in an ovine model^13^. As a comparison, another study using the same model analyzed the absorption of *Crotalus mictlantecuhtli* (previously *C. simus*) venom, which showed only 1.9% lymphatic absorption. This venom is dominated by larger enzymatic toxins, namely snake venom metalloproteases (SVMPs, 27% of total venom proteins) and snake venom serine proteases (SVSPs, 30% of total venom proteins), but also has smaller toxins, such as PLA_2_s (22.4% of total venom proteins), 14.3% of these are a neurotoxic PLA_2_ (crotoxin)^45,46^. When the venom components were analyzed separately, the results showed that after 12 h, the SVMPs reached the bloodstream in low proportions (7% of the injected dose of SVMPs), and the lymphatic absorption was 0.4%. For SVSPs, 8% was absorbed into the bloodstream after 12 h, and lymphatic uptake was 1%. Finally, for the smaller crotoxin, absorption could not be calculated because it was rapidly cleared from the bloodstream, leaving no detectable levels behind. However, crotoxin was still detectable in the lymph, where its absorption was low but consistent over time, likely due to a phenomenon known as the depot effect. In this process, the injection site acts as a reservoir, gradually releasing venom components over time, even after the initially absorbed venom has been cleared from the bloodstream^13,36^, as observed both in patients bitten by rattlesnakes and in an ovine model^47^. This process prolongs the presence of venom components in the bloodstream and the body, which may extend toxic effects, cause recurrence of envenoming, and generally complicate treatment^41,47,48^.

Apart from the size of the toxin molecules, molecular interactions, such as binding to or enzymatic degradation of extracellular matrix proteins or cellular components, can significantly alter the absorption pathways of toxins. Absorption may be changed substantially by the local tissue damage caused by such effects. For example, SVMPs in viper venoms generate severe lesions and hemorrhages in tissues^49–51^, which alter the integrity of both blood and lymphatic vessels and therefore may modify the absorption pattern^36,52^, particularly through the lymphatic system. When it comes to the elimination of the toxin molecules from the body, animal studies have shown that the elimination phase can take between 0.8 and 28 h, depending on the venom and species. In humans, an average elimination half-life of approximately 9.7 ± 1.3 h has been reported^42^.

In summary, the complexity of snake venoms and the diversity of their different components make the prediction of toxicokinetics complicated and highlight the necessity of experimental studies to understand the specific dynamics of venom absorption, distribution, and elimination^40^ (Table 1). In this context, large animal models offer particular value, as their physiological scale enables more clinically relevant assessment of lymphatic transport, depot effects, and systemic exposure than is feasible in small animal models. Improved understanding of venom toxicokinetics will be fundamental in developing strategies for dosing of antibody therapies and small molecule therapies that target specific toxins within venoms.

**Table 1:** Predicted translatability value of selected animal models for toxicokinetics, pharmacokinetics, pathophysiology, and treatment in snakebite research. Values are based on prior envenoming studies and knowledge of animal model physiology. ◯: Low translatability value; **◑**: Useful for initial screening ⬤: Direct translatability value

	Murine	Ovine	Porcine
**Toxicokinetics**^13^			
Venom absorption/distribution/elimination pattern	**◑**	**⬤**	**⬤**
Direct blood absorption	**◑**	**⬤**	**⬤**
Lymphatic absorption	◯	**⬤**	**⬤**
Depot effect	◯	**⬤**	**⬤**
**Pharmacokinetics**^75–77,86^			
Tissue penetration	**◑**	**⬤**	**⬤**
Clearance pathway	**◑**	**⬤**	**⬤**
Half-life in circulation	**◑**	**⬤**	**⬤**
**Pathophysiology**^14,77,79,81^			
Cardiovascular system	◯	**⬤**	**⬤**
Plasma volume	◯	**⬤**	**⬤**
Heart rate	◯	**⬤**	**⬤**
Thoracic duct cannulation	◯	**⬤**	**⬤**
Musculoskeletal system	**◑**	**⬤**	**⬤**
Wound healing	**◑**	**◑**	**⬤**
Skin structure	◯	**◑**	**⬤**
**Treatment**^14,82,83,88^			
Efficacy	**⬤**	**⬤**	**⬤**
Administration route	**◑**	**⬤**	**⬤**
Dosing	**◑**	**⬤**	**⬤**
Local tissue damage	**◑**	**◑**	**⬤**
Pressure immobilization	◯	**⬤**	**⬤**
Fasciotomy	◯	**⬤**	**⬤**
Drug toxicology	**◑**	**⬤**	**⬤**

## Pharmacokinetics of antibodies

In the treatment of snakebite envenoming, it is crucial to consider the pharmacokinetics of toxin-neutralizing molecules alongside the toxicokinetics of the venom components and potential depot effects involved in many snakebite cases^3,41,53,54^, as the pairing of pharmacokinetics with toxicokinetics will partly determine the efficacy of the treatment in a clinical setting.

The pharmacokinetics of antibodies, including both immunoglobulins (IgGs) and antibody fragments, are dependent on their ADME^55,56^. The absorption will depend on the route of administration, and most antibodies are given intravenously^55^. This allows rapid delivery of antibodies to the systemic circulation, delivery of large volumes, and complete systemic availability, ensuring a high systemic concentration. With respect to distribution, antibodies can reach and penetrate tissues from the circulation through extravasation, where the antibody is transported from the blood vessels to the tissue, either with fluid, via diffusion, or to some extent via fluid-phase endocytosis, or, for IgGs, also via receptor-mediated endocytosis^57^. Due to their size, larger antibody formats, such as IgGs and F(ab’)_2_s, penetrate tissue more slowly and stay longer in the bloodstream compared to smaller antibody formats, such as V_H_Hs, that distribute rapidly both into and within tissues^58^. Antibodies are eliminated either through catabolism by hepatic and reticuloendothelial pathways or by renal filtration when their molecular size is below the glomerular filtration threshold. The relative contribution of these pathways is primarily dictated by the size of the antibody molecule^59^. Hepatic/reticuloendothelial pathways primarily remove IgGs and F(ab´)_2_, while smaller antibody formats, such as Fabs and V_H_Hs, undergo renal filtration and are removed quickly from the circulation^60^.

For IgGs, their long plasma half-life is not only a result of their size, which prevents renal filtration, but also due to their Fc-domain that binds to the neonatal Fc receptor (FcRn) inside endosomes during endocytosis, which rescues the IgGs from lysosomal degradation and allows them to be recycled back into circulation^61^. In contrast, while F(ab’)_2_s are also not excreted through renal filtration, these fragments lack an Fc-domain and therefore cannot bind FcRn and be recycled, which results in a shorter half-life compared to IgGs^61^. To prolong the half-life and thereby reduce dosage, different antibody formats can be engineered in various ways^62^. For IgGs, this includes introducing mutations in the Fc-region to increase the affinity for FcRn^63^. For V_H_Hs, these can be fused to a (human) Fc-domain, linked to albumin, or made into di- or trivalent constructs with an albumin-targeting V_H_H to enable recycling via FcRn binding^60^. In addition, the half-life of F(ab’)_2_, Fabs, and V_H_Hs can be extended through PEGylation^64,65^. This will increase their size and reduce renal excretion, but also, to some extent, shield the antibodies from degradation by proteolytic enzymes in the bloodstream and recognition by the immune system and clearance by e.g., macrophages.

For small molecule inhibitors, which may be administered orally and rely on enteral absorption, efficacy will depend on their oral bioavailability as well as subsequent distribution, metabolism, and elimination, similar to antibody-based therapeutics^66^. Oral dosing offers flexibility, as treatment can be initiated before or during hospital care and repeated multiple times, potentially allowing cumulative or enhanced effects beyond those of a single administration. In addition, all pharmacokinetic parameters may be altered in the presence of hypotension or neurotoxicity following envenoming^67,68^.

For envenoming therapy in general, it may be relevant to ensure that antibodies or small molecule inhibitors are present in the bloodstream long enough for them to be able to neutralize the toxins that are released over time (e.g., due to the venom depot effect). Purely for the duration of effect, the relatively long half-life of the antibodies might be an advantage. On the other hand, increasing antibody size to extend their half-life may reduce their ability to rapidly penetrate deep tissues. In contrast, smaller molecules with greater tissue penetration may enable toxin neutralization even before they reach the circulation.

In summary, different therapeutic formats exhibit distinct pharmacokinetic properties (Table 2), and studies in large animal models can offer valuable insights into optimizing recombinant antibody-based or small molecule-based treatments for snakebite envenoming.

**Table 2:** Approximate half-life, clearance pathways, and tissue penetration properties of different antibody formats and selected small molecule inhibitors, based on representative preclinical and clinical studies across species^47,55–61,91,92^.

Antibody format	Molecular weight (Da)	Tissue penetration	Main route of clearance	Half-life in circulation
IgG	150,000	Low (mainly vascular) V_d_: 0.04–0.08 L/kg	FcRn-mediated recycling and hepatic/reticuloendothelial pathway	~21 days
F(ab)’_2_	100,000	Moderate V_d_: 0.2–0.4 L/kg	Hepatic/reticuloendothelial pathway	1–2 days
Fab	50,000	Moderate V_d_: 0.3–0.5 L/kg	Renal excretion	12–24 h
V_H_H	15,000	High (deep tissue) V_d_: 1–2 L/kg	Renal excretion	1–2 h
Marimastat^91^	331	N/A	Renal excretion	8–10 h
Varespladib^92^	380	N/A	Renal excretion	5 h

V_d_ volume of distribution. N/A. Not available.

## Large animal models used in the study of snakebite envenoming

Historically, envenoming research has been conducted using primarily small animal models, including mice, rats, guinea pigs, rabbits^69^, cats^70^, dogs, and others. These have provided the basis of our knowledge of the pathophysiology of diverse venoms and isolated toxins and have allowed the evaluation of antivenoms and other neutralizing molecules. Large animal models in envenoming research have included sheep, goats, pigs, and non-human primates^71^, among others. While non-human primates have been described to be good research models due to their close phylogenetic relation with humans^72^, today they are rarely used for envenoming research due to ethical, regulatory, and logistical constraints^73,74^. Horses and camelids are extensively used for the production of plasma-derived antivenoms and the generation of nanobody libraries, respectively. However, their use as models for human physiology in envenoming has been very limited, and therefore, they were considered outside the scope of this review.

Sheep have been instrumental in the field of snakebite envenoming, where they have been used to study the toxicokinetics of venoms and the pharmacokinetics of antivenoms^40,75,76^. The reason for choosing sheep includes their ease of handling as a farm animal and that they are a relatively low-cost large animal model compared to other large animals, such as non-human primates. They have cardiovascular systems that are comparable to those of humans, with similar plasma volumes ( ~40 mL/kg) and heart rates (50–100 beats per minute), although their maximum heart rate is significantly higher (280 vs. 150 beats per minute in humans)^77^. Also, thanks to the availability of thoracic duct cannulation protocols, sheep are frequently used in lymphatic research, making them valuable for studying venom absorption during snakebite envenoming^77,78^ (Table 1).

Porcine models have been widely used in biomedical research due to their anatomical and physiological similarity to humans. They have served as models for studying the human musculoskeletal system, wound healing, and cardiovascular function^79,80^. Juvenile pigs have also been proposed as a translatable model for pediatric drug development^80^. In venom research, fundamental differences in murine skin anatomy make it very hard to study the local effects of envenoming in mice^81^. Instead, pigs have proven particularly valuable for investigating these local effects, such as dermonecrosis, due to the structural and functional resemblance of their skin to human skin^79^. These similarities include epidermal thickness (70 –120 µm) and cutaneous vascular anatomy^79^. The porcine model has also been used to study venom-induced increase in intramuscular pressure (compartment syndrome)^82^, evaluate antivenom efficacy and administration routes^83^, and test techniques for preventing and treating local tissue damage, such as pressure immobilization and surgical cut of fascia surrounding muscles to relieve pressure (fasciotomy)^82,84^, in addition to other applications. For example, using a domestic pig model, the repurposed drug varespladib, a small molecule inhibitor of PLA_2_s, was observed to successfully revert otherwise lethal envenoming by two predominantly neurotoxic elapid snake venoms when administered orally and intravenously^33,85^. The potential of this small molecule inhibitor to be used as an oral drug in clinical practice is supported by existing data on how various drug formulations move through the digestive system in pigs^86^. Another example is the porcine model of *Deinagkistrodon acutus* envenoming, developed by Lai and collaborators, which provides a foundation for the future evaluation of novel therapeutic strategies against *D. acutus* venom^14^.

**Fig. 1:**
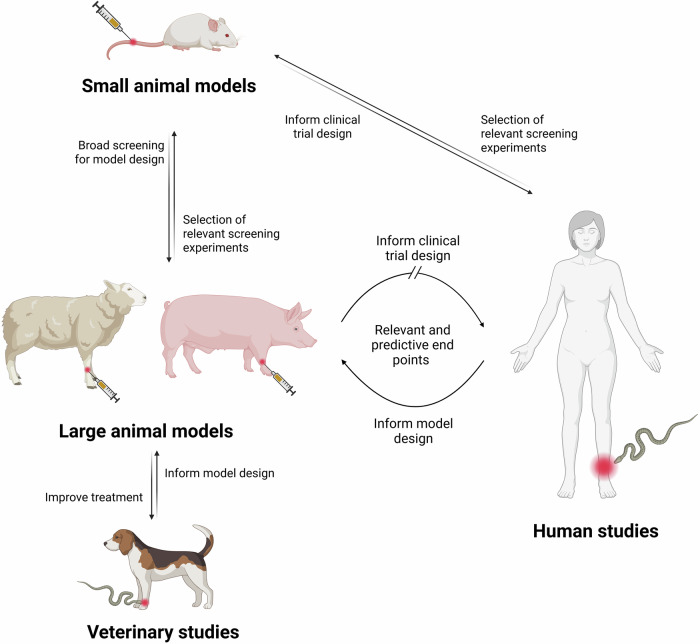
Translational role of large animal models for developing snakebite envenoming treatments. While small animal models provide a tool for initial and broad screening, large animal models provide predictive endpoints that support the design and optimization of clinical trials and therapeutic strategies in humans. In turn, clinical data and case reports (both human and veterinary) can refine the selection of relevant endpoints in animal studies, creating a reciprocal and iterative translational cycle. This bidirectional feedback loop strengthens both preclinical modeling and patient care. Identifying and addressing knowledge gaps in this cycle is essential for translating novel snakebite therapeutics into clinical practice. Created using BioRender.

Compared to small animal models (e.g., mice or rabbits), experiments in large animals pose significant challenges: they are costly, require specialized veterinary personnel, proper surgical facilities, and the acquisition of strict(er) ethical permits^36^. Therefore, the gold standard murine model is still relevant, especially during initial screening stages to identify the main toxic effects of venoms or to evaluate the overall neutralization capacity of a treatment. Yet, their limited size restricts pharmacokinetic and clinically relevant assessments. Together, small and large animal studies provide complementary information from discovery to clinical application (Fig. 1).

The use of common livestock and farm animals can help mitigate some of these issues, since protocols for their handling and veterinary care are readily available, and there is a comprehensive understanding of their anatomy and physiology. Often, available knowledge from snakebite case reports also exists, which can be of great value when comparing experimental envenoming with real-life cases and allows for the identification of relevant endpoints to design the experiments^35,87^. Continued research in this area is valuable, as identifying more relevant and predictive endpoints can improve the quality and translational value of i*n vivo* studies. This can contribute to refining experimental design, reducing the number of animals required, and facilitating the development of robust in vitro alternatives where applicable^88^.

## Translation of promising new molecules into feasible clinical treatments

Preclinical research using large animal models is informative in bridging promising snakebite therapeutics to human application by providing key data on efficacy, pharmacokinetics, and drug toxicology. As previously mentioned, the small molecule varespladib was evaluated in pilot studies using juvenile pigs and was shown to reverse the neurotoxic effects of venom from the Eastern coral snake (*M. fulvius*)^85^ and taipans (*Oxyuranus scutellatus*) from Australia and Papua New Guinea, after 45 min delayed administration^33^. Varespladib has now completed Phase 2 clinical trials in India and the USA. These trials have shown a potential benefit of varespladib treatment when initiated within 5 h after the snakebite^89^, although there was no conclusive benefit observed for the defined primary outcome of the clinical trial.

Because of their larger size, which permits the study of more clinically relevant pharmacokinetics, large animal models can help refine therapeutic dosing, inform dose interval recommendations when necessary, and support the development of new treatment protocols^33^. This can streamline regulatory approval, as regulatory agencies may offer accelerated pathways or adaptive trial designs when compelling large animal data is available. This is especially valuable for rare or neglected conditions like snakebite envenoming, where human clinical trials may be logistically challenging and/or ethically complex to conduct.

Clinical reports and studies of snakebite envenoming are essential for determining the relevance of large animal models and for the selection of endpoints to design informative clinical trials. To this end, detailed clinical observations of snakebite envenoming progression, including organ dysfunction and patient outcomes, can offer critical data points. By aligning large animal model endpoints with human clinical parameters, for example, tissue damage biomarkers, organ function monitoring, and time-to-recovery metrics, researchers can increase the predictive value of animal studies. However, establishing clear and measurable endpoints in snakebite envenoming is challenging, given the wide variability in pathologies induced by venoms from the diverse array of snake species worldwide^90^. In addition, the study of the natural history of envenoming in humans is hindered by the medical and ethical requirement for prompt use of antivenom, the unknown venom doses involved, variation in subject health, specific bite site anatomy, and the heterogeneity of the clinical course. Given this variability and the limited availability of detailed clinical data, small animal models play an important role as a first screening approach. They help to identify the most relevant experiments and endpoints to select for each type of envenoming, thereby guiding the design of more informative large animal studies.

At present, few novel molecules for snakebite treatment have progressed from large animal studies to clinical application; however, ongoing efforts aim to better define envenoming doses and pathology in these models. As mentioned, one such candidate is varespladib^85,89^. However, the translation of new molecules for snakebite treatment from large animal models to the clinic is still in the early stages of development.

## Concluding remarks

To facilitate the development of new and better treatments for human victims of snakebite envenoming, a comprehensive understanding of both the pharmacokinetics of neutralizing molecules and the toxicokinetics of venom components is needed. To this end, large animal models can provide valuable insight since they offer a better physiological comparison to humans than small animal models, such as mice, which are typically used in early preclinical testing. In addition to testing efficacy, large animal models may enable more accurate dose predictions and drug toxicology assessments. However, there is a significant gap in correlating data from large animal studies with clinical outcomes in human snakebite victims. To overcome this gap, systematic studies that align preclinical findings with real-world patient responses are needed. Furthermore, standardized endpoints and clear guidelines on the design and interpretation of large animal experiments would facilitate the clinical translation of both traditional plasma-derived antivenom and new envenoming therapies, such as recombinant antivenoms and small molecule inhibitors, and accelerate their development. In turn, such efforts will likely both support regulatory approval and contribute to more effective and safer therapies for snakebite victims globally.

## Data Availability

No datasets were generated or analyzed during the current study.
